# ParA and ParB coordinate chromosome segregation with cell elongation and division during *Streptomyces* sporulation

**DOI:** 10.1098/rsob.150263

**Published:** 2016-04-27

**Authors:** Magdalena Donczew, Paweł Mackiewicz, Agnieszka Wróbel, Klas Flärdh, Jolanta Zakrzewska-Czerwińska, Dagmara Jakimowicz

**Affiliations:** 1 Faculty of Biotechnology, University of Wrocław, Joliot-Curie 14A, Wrocław 50-383, Poland; 2 Department of Biology, Lund University, Sölvegatan 35, Lund 22362, Sweden; 3 Ludwik Hirszfeld Institute of Immunology and Experimental Therapy, Polish Academy of Sciences, Weigla 12, Wrocław 53-114, Poland

**Keywords:** chromosome segregation, bacterial cell division, bacterial cell growth, *Streptomyces*, sporulation, ParAB

## Abstract

In unicellular bacteria, the ParA and ParB proteins segregate chromosomes and coordinate this process with cell division and chromosome replication. During sporulation of mycelial *Streptomyces*, ParA and ParB uniformly distribute multiple chromosomes along the filamentous sporogenic hyphal compartment, which then differentiates into a chain of unigenomic spores. However, chromosome segregation must be coordinated with cell elongation and multiple divisions. Here, we addressed the question of whether ParA and ParB are involved in the synchronization of cell-cycle processes during sporulation in *Streptomyces*. To answer this question, we used time-lapse microscopy, which allows the monitoring of growth and division of single sporogenic hyphae. We showed that sporogenic hyphae stop extending at the time of ParA accumulation and Z-ring formation. We demonstrated that both ParA and ParB affect the rate of hyphal extension. Additionally, we showed that ParA promotes the formation of massive nucleoprotein complexes by ParB. We also showed that FtsZ ring assembly is affected by the ParB protein and/or unsegregated DNA. Our results indicate the existence of a checkpoint between the extension and septation of sporogenic hyphae that involves the ParA and ParB proteins.

## 1. Introduction

Robust cell proliferation requires precise coordination of key cell-cycle processes. In most rod-shaped bacteria, chromosome replication and segregation occur during longitudinal cell elongation and are shortly followed by cell division. Active chromosome segregation begins soon after the initiation of replication in the *oriC* region (origin of replication). The ParAB–*parS* system mediates the migration of newly replicated *oriCs* either unidirectionally (e.g. in *Caulobacter crescentus*) or bidirectionally (e.g. in vegetatively growing *Bacillus subtilis*) towards the cell pole(s) [1]. Nucleoprotein complexes named segrosomes are formed by ParB binding to *parS* sites, which are usually scattered in the proximity of the *oriC* region [2–5]. The movement of the segrosomes requires an interaction between ParB and ParA as well as the ATPase activity of ParA, its dimerization and interaction with DNA and/or polymerization [6–8]. Interestingly, *parAB* deletion is associated with diverse phenotypes in different bacterial species; in *C. crescentus* the genes are essential, but in *B. subtilis* their deletion has only a minor effect on the growth. The explanation for these differences is that ParAB proteins are involved in the coordination of chromosome segregation with different cell-cycle processes, such as chromosome replication (in *B. subtilis* and *Vibrio cholerae*), cell growth and/or cell division (*C. crescentus*, *Mycobacterium smegmatis* and *Streptomyces coelicolor*) [9–13]. Thus, the cell-cycle coordinating mechanisms involving ParAB appear to be specific to particular bacterial species.

Chromosome segregation must be synchronized with the formation of the division septum, which separates the two daughter cells. The septation starts with the polymerization of FtsZ into a ring structure (Z-ring) that determines the division plane [7,14]. In model rod-shaped bacteria (*Escherichia coli*, *B. subtilis*), two systems, Min and nucleoid occlusion (NO), prevent guillotining of the unsegregated chromosomes by the division septum [15,16]. Both systems use inhibitors of FtsZ polymerization (MinC and Slm in *E. coli* or Noc in *B. subtilis*) that control the timing and positioning of the Z-ring formation [17]. However, these systems are not universal in all bacteria. Many bacteria, such as those belonging to the Actinobacteria, do not appear to possess homologues of Min or NO proteins [18]. The spatio-temporal control of cell division in these bacteria remains to be elucidated.

In mycelial *Streptomyces*, a member of the phylum Actinobacteria, chromosome segregation and cell division, followed by cell separation, occur exclusively during sporulation. Unlike model rod-shaped bacteria, *Streptomyces* form hyphae, which extend apically and form branches. During vegetative growth, chromosomes remain uncondensed and their replication is not followed by cell division [19]. This mode of growth leads to the formation of elongated multigenomic compartments that are not physically separated, although they are delimited by rare septa. Interestingly, cell division is not essential for *S. coelicolor* vegetative growth [20–22], but it is crucial for sporulation. A sporulating colony produces multigenomic sporogenic hyphae, named aerial hyphae, which subsequently transform into chains of unigenomic exospores [19]. The conversion of hyphae to spore chains involves synchronized, multiple septation accompanied by the condensation and segregation of tens of chromosomes into prespore compartments. In aerial hyphae, FtsZ forms a ladder-like array of regularly spaced Z-rings along the hyphal compartment [23,24]. Synchronized septation imposes special requirements on the coordination of Z-ring positioning with multiple chromosome segregation in *Streptomyces*. As mentioned above, *Streptomyces* do not have homologues of Min proteins and nucleoid occlusion proteins (Noc and SlmA). Recent experiments indicated that in *S. coelicolor*, FstZ is recruited to the septum site by SsgB, which, unlike the system based on Min and NO, is an example of positive control of septal position [25]. However, it is still not known if a negative control mechanism(s) of septum positioning is also present in *Streptomyces*.

In *Streptomyces*, as in other rod-shaped bacteria, the ParAB proteins mediate segregation of multiple chromosomes in the sporogenic hyphal compartment [26,27]. The *parA*B operon (similarly to *ftsZ* [28]) is under the control of developmentally regulated promoters, which are strongly induced in sporulating hyphae [29]. In vegetative hyphae of *S. coelicolor*, both proteins are mostly associated with the tips, while during sporulation ParA extends along the hyphae, and ParB forms an array of regularly spaced complexes [26,30]. In the septated hyphae, each prespore contains a single ParB complex, positioning the chromosome between nascent septa. The elimination of ParA and/or ParB from *S. coelicolor* disturbs chromosome segregation and leads to the formation of anucleate spores [30]. ParAB deletions also affect sporulation septation, suggesting that the distribution of chromosomes determines septa positioning [30]. Interestingly, chromosome segregation and septation are also affected by modified DNA supercoiling, which can be induced by topoisomerase I (TopA) depletion [31]. Moreover, TopA assists in the regular distribution of segrosomes. The aberration in segrosome separation observed in a TopA depletion strain is associated with complete blockage of cell division [31]. Interestingly, moderate TopA depletion increased DNA compaction and did not inhibit septation but affected the distance between the septa. The aberrant septation in strains with chromosome missegregation suggests a role for the nucleoid in the regulation of septa placement in *Streptomyces*.

The ParAB system in *S. coelicolor* was also reported to be involved in the control of tip extension based on the interaction between ParA and Scy [11,32]. Scy is a tip-associated protein involved in polar growth. It also affects ParA polymerization and localization, while ParA presumably influences the Scy complex assembly. This idea is supported by our observation that the sporogenic hyphae of the *S. coelicolor parA* deletion strain are elongated. Although the analysis did not clarify whether *parA* deletion extends the time of hyphal growth and delays sporulation or affects the rate of hyphal elongation, the dynamic interplay between ParA and Scy at the tips of hyphae was suggested to provide the molecular switch from aerial hyphal extension to sporulation [11].

Thus, previous studies of *Streptomyces* sporulation have indicated that ParAB proteins play an important role in synchronizing the events during aerial hyphae sporulation. These studies suggested that ParA is involved in the control of sporogenic hyphal extension. They also indicated that by determining the position of nucleoids, segregation proteins affect the placement of the septa. This suggests a negative regulatory mechanism controlling cell division. However, those studies were performed using *S. coelicolor* as the model species. *S. coelicolor* aerial hyphae develop exclusively at the interface between medium and air, which impedes (due to impaired sporulation at the interface between agar and coverslip) live cell time-lapse imaging of the whole differentiation process, from the emergence of sporogenic hyphae to the separation of spores [33]. Here, to verify the hypotheses that ParA and ParB have a role in coordination of sporulation events, we used real-time single cell analysis of differentiation, including analyses of hyphal growth, division and chromosome segregation, in a novel model species, *Streptomyces venezuelae*. An advantage of *S. venezuelae* as a model species for studies of differentiation is its ability to sporulate in liquid culture (and under the coverslip) [34]. Fluorescence time-lapse microscopy enabled us to precisely measure the time of hypha elongation and sporulation as well as to determine the timing of ParA and FtsZ appearance during differentiation. We showed that segregation proteins control hyphae elongation and division in *Streptomyces*.

## 2. Material and methods

### 2.1. DNA manipulations, bacterial strains and growth conditions

DNA manipulation, culture conditions, antibiotic concentrations and conjugation or transformation methods followed standard procedures for *Escherichia coli* [35] and *Streptomyces* [36]. *E. coli* and *S. venezuelae* strains used in this study are listed in the electronic supplementary material, table S1. *S. venezuelae* was cultivated in MYM liquid medium and on MYM agar plates supplemented with 200 µl of trace element solution per 100 ml [36].

### 2.2. Construction of *Streptomyces venezuelae* mutant strains

*Streptomyces venezuelae* mutants were constructed using PCR targeting (electronic supplementary material, table S2), similar to the method described for *S. coelicolor* [37]. For strain construction details, see the electronic supplementary material. Conjugation from *E. coli* to *S. venezuelae* was performed as previously described [38]. All modified strains were verified by PCR and Southern and western blotting analyses to confirm gene deletions.

### 2.3. Microscopy and image analysis

For microscopic observations, strains were grown for 16–18 h on coverslips inserted in minimal solid medium (MM) supplemented with 1% mannitol. Sample preparation for fluorescence microscopy was performed as described previously [30]. For DNA visualization, samples were incubated for 1 h at room temperature with DAPI and with WGA-Texas Red for cell wall staining. For time-lapse imaging, spore dilutions were spotted onto cellophane membranes on MM solid medium supplemented with 1% mannitol and cultured for 24–48 h before the start of the experiment. The cellophane membrane was transferred to a µ-dish (*Ø*35 mm, Ibidi) and covered with a block of agar [33]. To maintain a temperature of 30°C during the time-lapse experiments, the µ-dish was placed on the microscope stage in a temperature-controlled chamber. Fluorescence microscopy was carried out using a Zeiss Observer Z1 inverted microscope equipped with a Plan-Neofluar objective 100×/1.30 Oil and AxioCam MRm Camera. The Definite Focus feature was used to maintain the same focal position throughout the time course. Images were acquired using differential interference contrast (DIC) and fluorescence channels: EGFP (EX BP 470/40, BS FT 495, EM BP 525/50), DAPI (EX G 365, BS FT 395, EM BP 445/50), YFP (EX BP 500/25, BS FT 515, EM BP 535/30) and DsRed (EX BP 550/25, BS FT 570, EM BP 605/70). Images were acquired every 15 min using DIC and EGFP/YPet with exposure times of 10 and 1000 ms, respectively. Images were analysed using AXIOVISION or ZEN software (Zeiss). In the time-lapse analyses, approximately 30 hyphae were analysed from at least four independent experiments. Care was taken to ensure that the experiments using different strains were performed under the same conditions.

The intensity of EGFP and YPet fluorescence was measured using the ‘profile’ function of ZEN 2012 software. A straight line was placed along the hyphae length. The corresponding background fluorescence was subtracted from the fluorescence measured for each hypha. The increase of the FtsZ-YPet and ParA-EGFP fluorescence was detected when the average value of the fluorescence intensity along the hyphae increased more than 10% over the fluorescence intensity in the same hyphae at earlier time points. The Z-ring fluorescence intensity was calculated as an average of the seven highest peaks present in the fluorescence profile (referring to the seven most representative Z-rings). Statistical analyses were performed using Statistica (StatSoft Inc. 2011, v. 10, www.statsoft.com). Because the assumption of normal distribution was violated, the non-parametric counterpart of ANOVA, the Kruskal–Wallis test, was applied for the comparisons of FtsZ-YPet fluorescence and parameters describing hyphae growth and length in the *Streptomyces* strains. For comparison of the TopA-depleted strain with the wild-type strain, unpaired Student's *t*-tests and Mann–Whitney tests were applied accordingly. Differences were considered significant when *p*-values were lower than 0.05. The average growth rate of each hypha was calculated as the slope of a line fitted to the relationship between the hypha length and growth time.

Super-resolution three-dimensional (3D)-SIM imaging was performed with a V3 DeltaVision OMX 3D-SIM Blaze system (Applied Precision/GE Healthcare) equipped with a ×60/1.42 oil UPlanSApo objective (Olympus), 405 nm and 488 nm diode lasers and three sCMOS cameras (PCO). Each 3D-SIM stack was composed of 225 images (512 × 512 pixels) consisting of 15 *z*-sections (125 nm *z*-distance), with 15 images per *z*-section, the striped illumination pattern rotated to three angles (−60°, 0°, +60°) and shifted in five-phase steps. Acquisition settings were 100 ms exposure with a 488 nm laser (attenuated to 100% transmission). The reconstruction of 3D-SIM raw data were performed with SoftWoRx 6.0 (Applied Precision) using a Wiener filter setting of 0.002. Reconstructed image-stacks were 3D rendered and visualized using Softworks and ImageJ software. The fluorescence intensity along the Z-rings was measured using the profile function in ImageJ software.

### 2.4. Chromatin immunoprecipitation and bioinformatics analysis

Chromatin immunoprecipitation assays followed by sequencing (ChIP-seq) were performed as previously described by Al-Bassam *et al*. [39] with minor changes. Each strain was cultured in MYM medium supplemented with trace elements for an appropriate time (14 and 20 h for the wild-type, Δ*parA*, Δ*parB::apra* and Δ*parAB* strains; 30 h for the TopA depletion mutant). After cross-linking (formaldehyde added to a final concentration of 1% (v/v)) and lysis, the samples were sonicated for six to seven cycles (20 s each) to shear the chromosome into fragments ranging from 200 to 600 bp. The samples were centrifuged and pre-cleaned with Protein A Sepharose (Sigma). Then, 2 µg of purified polyclonal anti-ParB antibodies was added to each cell lysate, and the mixtures were incubated on a rotating wheel at 4°C overnight. Protein A Sepharose was added to cell lysates and incubated for the next 4 h. The samples were centrifuged, and the pellets were washed with IP buffer (50 mM Tris–HCl pH 8.0, 250 mM NaCl, 0.80% Triton, protease inhibitors) and eluted by overnight incubation at 65°C with IP elution buffer (50 mM Tris–HCl pH 7.6, 10 mM EDTA, 1% SDS). After centrifugation, the pellets were re-extracted with TE buffer (50 mM Tris–HCl pH 8.0, 10 mM EDTA), incubated with proteinase K (Roche) for 1.5 h at 55°C and extracted twice with phenol and once with chloroform. Finally, samples were purified with QiaQuick columns (Qiagen). For each immunoprecipitation sample, we used its corresponding input control of total DNA. DNA concentration was quantified using a NanoDrop spectrophotometer (ThermoScientific). Libraries were constructed and sequenced by the Karlsruher Institut für Technologie (KIT). Experiments were performed in duplicate.

ChiP-seq sequence data were aligned (mapped) to the *S. venezuelae* chromosome using two aligners, Bowtie 1.1.1 and Novoalign 3.02.08 (Novocraft Technologies Sdn Bhd, www.novocraft.com) [40,41]. With Bowtie, we selected options reporting only alignments in the best alignment ‘stratum’, that is, having the least number of mismatches, which was assumed to be one up to three. Using Novoalign, we applied a method reporting all alignment locations. SAMtools 1.1 and BEDTools 2.17 were used to manipulate the obtained files and convert formats [42]. To find peaks, that is, regions with significant numbers of mapped reads, we used Peak Finder MetaServer 1.3 (PFMS), which collects results from several peak finders and produces consensus peaks [43]. We applied three recommended peak finders: MACS 1.4.2 [44], CisGenome 2.0 [45] and SISSRs 1.4 [46]. Finally, Integrative Genomics Viewer (IGV) 2.3.40 [47] was used to visualize data.

To search *Streptomyces* genomes for potential *parS* sequences, we constructed a position weight matrix (PWM) based on sequences (GTTTCACCTGAAAC) identified experimentally in *S. coelicolor* by Jakimowicz *et al*. [26]. Then, the PWM was used by the Motif Occurrence Detection Suite (MOODS), 1.0.2 [48] to find corresponding sequences in the *Streptomyces* chromosomes, which were downloaded from the GenBank database under accession numbers NC_003888 (*S. coelicolor*) and NC_018750 (*S. venezuelae*). The DNA walk method was used to visualize the A + T content along the chromosome [49].

## 3. Results

### 3.1. Sporogenic hyphal growth stops at the time of Z-rings formation and ParA accumulation along the hyphae

Previous reports have indicated that the extension of the sporogenic hyphae stops before the commencement of further stages of development, that is, chromosome condensation, segregation and hyphae septation [50]. The switch from the extension of aerial hyphae to the formation of spores was suggested to be mediated by ParA [11]. To verify this hypothesis, we used the novel model organism, *S. venezuelae*, in which sporulation is not limited to the interface between solid medium and air [34]. Therefore, we could use single cell time-lapse microscopy to monitor the whole sporulation process in detail.

**Figure 1.**
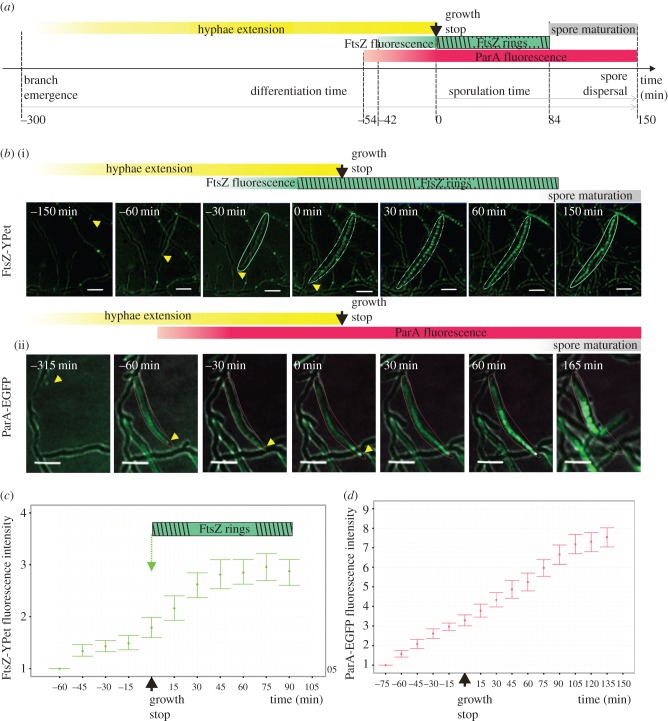
Timing of sporogenic hyphae extension, FtsZ ring formation and ParA accumulation during *S. venezuelae* sporulation. (*a*) Scheme of hyphae differentiation stages. (*b*) Time-lapse snapshots showing examples of hyphae in particular stages of development with FtsZ-YPet fluorescence (green) merged with the DIC image (i) or ParA-EGFP (green) merged with the DIC image (ii). The yellow arrowhead marks the position of growing hyphae, the green outline indicates the hyphae with an increase of FtsZ fluorescence, the broken green outline indicates the hyphae with Z-rings and the red contour shows the hyphae with increased ParA-EGFP fluorescence. Scale bars, 5 µm. (*c*) Changes in FtsZ-YPet fluorescence intensity (fold increase over the fluorescence intensity at earlier time points, measured for 35 hyphae from at least four independent experiments) during sporulation. (*d*) Changes in ParA-EGFP fluorescence intensity (fold increase over the fluorescence intensity at earlier time points, measured for 30 hyphae) during sporulation.

The *S. venezuelae* merodiploid reporter strain expressing *ftsZ-ypet* (from the native promoter) as a marker of Z-rings (MD100) was used to monitor the timing of sporulation septation in relation to the extension of the sporogenic aerial hyphae. Growth of this strain was similar to the growth of the wild-type parent strain (electronic supplementary material, figure S1), although its sporulation was slightly delayed. Images of the *S. venezuelae* sporulating hyphae were captured every 15 min for 16–20 h. Selected hyphae, which underwent the whole sporulation process and could be observed from branch emergence to the separation of individual spores, were analysed in subsequent images. These analyses enabled us to measure the parameters describing the growth and differentiation of sporogenic hyphae, such as the duration of hyphal extension (calculated as time from branch emergence to the cessation of its growth), the final length of sporulating hyphae and the hyphal tip extension rate. The appearance of Z-ring ladders was regarded as the initiation of septation, while the time period during which regular Z-rings were clearly detectable was considered the septation time. Additionally, we calculated the spore maturation time and the overall sporulation and differentiation time (figure 1*a*, tables 1 and 2).

**Table 1.** Basic parameters describing sporogenic hyphal growth in the studied *S. venezuelae* strains. The data are based on 35 hyphae from at least four independent experiments. The mean ± s.d. and the median (in parentheses) are shown; asterisks indicate statistically significant results in comparison with wild-type (*p < 0.1, **p < 0.01, ***p < 0.001).

parameter	WT (*ftsZ-ypet*)	Δ*parA* (*ftsZ-ypet*)	Δ*parB* (*ftsZ-ypet*)	Δ*parAB* (*ftsZ-ypet*)
time of sporogenic hyphae extension (min)	299±61 (285)	335±85 (315)	367±88 (370) *	319±115 (300)
length of sporogenic hyphae at growth cessation (µm)	36±13 (34)	61±16 (57) ***	31±11 (30)	31±12 (28)
rate of sporogenic hyphae extension (µm h^−1^)	7.6±2.2 (7.4)	12±3.5 (11.1) ***	5.5±2.1 (4.8) **	6.3±2.1 (6.2)

**Table 2.** Basic parameters describing FtsZ-YPet fluorescence and timing of Z-ring formation and disassembly in the studied *S. venezuelae* strains. The data are based on 30 hyphae from the TopA depletion strain and 35 from the other strains from at least four independent experiments. The mean ± s.d. and the median (in parentheses) are shown; asterisks indicate statistically significant results in comparison to wild-type (***p < 0.001).

parameter	WT (*ftsZ-ypet*)	Δ*parA* (*ftsZ-ypet*)	Δ*parB* (*ftsZ-ypet*)	Δ*parAB* (*ftsZ-ypet*)	TopA depl. (*ftsZ-ypet*)
time of FtsZ fluorescence appearance^a^ (min)	−42±7 (45)	−44±4 (45)	−43±6 (45)	−44±4 (45)	—
time of Z-ring appearance^a^ (min)	0±9 (0)	15±12 (15) ***	−28±15 (−30) ***	−39±12 (−30)***	−191±49 (−203) ***
lifetime of FtsZ rings (min)	84±26 (90)	79±12 (75) ***	117±21 (120) ***	135±28 (135)***	205±52 (210)***
time from Z-ring disappearance to spore separation (min)	63±37 (60)	60±17 (60)	62±27 (60)	47±20 (45)	26±10 (30) ***
total sporulation time^b^ (min)	150±51 (150)	154±21 (150)	151±38 (150)	144±29 (135)	38±11 (30) ***
total differentiation time^c^ (min)	449±82 (450)	489±89 (465)	518±108 (510)	461±125 (450)	372±77 (390)

^a^In relation to growth cessation.

^b^From growth cessation to spore separation.

^c^From emergence of sporogenic hyphae to spore separation.

**Figure 2.**
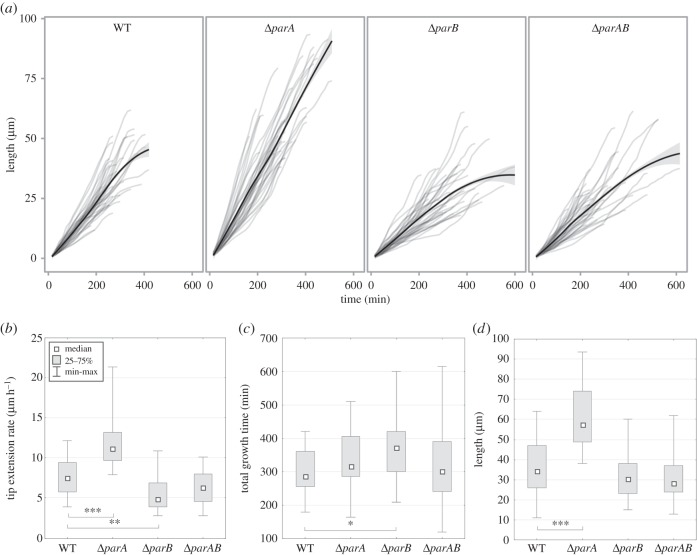
The effect of segregation proteins ParA and ParB on the extension of sporogenic hyphae. (*a*) Plot of hyphae length over time in the wild-type (WT), Δ*parA*, Δ*parB* and Δ*parAB* strains, (*b*) box plots for hyphal growth rate, (*c*) box plots for total growth time of the hyphae, (*d*) box plots for hyphal length (asterisks indicate statistically significant results in comparison with wild-type; *p < 0.1, **p < 0.01 and ***p < 0.001).

Sporulation starts with the extension of sporogenic hyphae, which subsequently undergo synchronized division. The sporogenic branches of the strain MD100 (*ftsZ-ypet*) extended for 299 ± 61 min (mean ± s.d.). The final length of the hyphae was 36 ± 13 µm (table 1, see also figure 2*a*). These measurements allowed us to calculate average hyphal extension rate (table 1), which was 7.6 ± 2.2 µm h^−1^. The extension rate was constant during the growth of the aerial hyphae as shown by the uniform increase of hyphal length at subsequent time points. Only at the beginning of aerial hyphal growth and close to growth cessation was the rate slightly lower than the growth stage. The increase in FtsZ-YPet fluorescence was detected in the MD100 sporogenic hyphae 42 ± 7 min prior to growth cessation (figure 1*b,c*; electronic supplementary material, movie S1). At first, FtsZ-YPet fluorescence was observed as dispersed fluorescence along the hyphae. The Z-rings became clearly visible at growth cessation (approx. 33 min after the increase in FtsZ-Ypet fluorescence) and remained noticeable for 84 ± 26 min. The position of the rings did not change, but their fluorescence increased slightly over 29 ± 17 min to reach the maximal level (more than three times higher than the initial fluorescence) and remained constant for the next 55 ± 16 min (figure 1*c*). Approximately 63 ± 37 min after the disappearance of the FtsZ rings, the spores rounded and separated (figure 1*a,b*, table 2; electronic supplementary material, movie S1). The total time of differentiation from the emergence of sporogenic hypha to spore separation was approximately 7.5 h (449 ± 82 min), of which 5 h were required for hyphae to reach the full length. The total time of sporulation measured from the cessation of hyphal growth to spore separation was 2.5 h, of which septation lasted approximately 1.5 h, followed by 1 h spore maturation. The timing of FtsZ ring formation in relation to the arrest of tip extension showed very little variation (table 2), indicating the precise coordination between those processes.

To determine the timing of chromosome segregation, we attempted to construct fluorescent protein fusions of the segregation proteins, ParA and ParB. In aerial hyphae, ParA extends along the hyphae to accompany ParB complexes only during chromosome segregation. The attempts to construct functional ParB-EGFP fusions were not successful, and the constructed strain had a chromosome segregation defect similar to the Δ*parB* strain. The strain expressing *parA*-*egfp* from the native chromosomal locus (MD004) exhibited slightly impaired chromosome segregation (5% anucleate spores in MD004 compared with 10% in the Δ*parA* strain and 0.4% in the wild-type strain) but septation disturbances, which are associated with *parA* deletion, were not detected in MD004 (electronic supplementary material, figure S1*b*). The disturbances in segregation may be due to the fusion of EGFP to ParA resulting in reduced protein dynamics/stability and/or the slightly lower ParB level in the MD004 strain in comparison to the wild-type strain (electronic supplementary material, figure S1*a*). The ParA-EGFP fluorescence was visible in MD004 as a diffuse bright signal extending from the tip along the hyphae (figure 1*b*(ii); electronic supplementary material, movie S2). The same localization was observed when immunofluorescence was used for ParA localization (electronic supplementary material, figure S2*a*). Interestingly, the tip-associated ParA complexes that were previously described in *S. coelicolor* [11,30] were not detected in *S. venezuelae*. The ParA fluorescence along the hyphae was detected 54 ± 14 min before the cessation of hyphal growth in all analysed hyphae, and its signal increased for the next 164 ± 5 min and then remained constant (figure 1*b*(ii), figure 1*d*; electronic supplementary material, movie S2). The fluorescence exceeded the initial intensity by approximately three times at growth cessation and at spore separation reached a level approximately eight times higher than the initial intensity (figure 1*d*). These results suggest that ParA starts to accumulate almost 1 h before growth cessation. Its increasing fluorescence was visible for approximately 2.5 h until spore separation.

In summary, our time-lapse analyses allowed determination of the growth parameters of developing *S. venezuelae* sporogenic hyphae. We showed that growth cessation is concurrent with Z-ring formation and the onset of chromosome segregation, which is marked by the accumulation of ParA proteins.

### 3.2. Segregation proteins affect the extension rate of sporogenic hyphae

A time-lapse analysis of the *S. venezuelae* sporulating hyphae confirmed that ParA appearance is associated with the cessation of hyphal growth. Our previous studies of *S. coelicolor* indicated that ParA affected hyphal extension [11,32]. To determine if the sporulation proteins influence the growth of *S. venezuelae* sporogenic hyphae, we analysed *parA*, *parB* and *parAB* deletion strains expressing *ftsZ-ypet* (MD111, MD021 and MD031, respectively). As observed previously in *S. coelicolor* [27,30], *parA*, *parB* and *parAB* deletions in *S. venezuelae* also resulted in chromosome missegregation and irregular septation during sporulation (restored in complemented strains, electronic supplementary material, figure S1*b*), although the disturbances were less pronounced than in *S. coelicolor*.

The elimination of ParA in *S. venezuelae* led to an increased rate of tip extension in the sporogenic hyphae to approximately 160% of the wild-type rate (figure 2*a*). A time-lapse analysis of the *parA* deletion strain (MD111) growth revealed that its growth rate was 12.0 ± 3.5 µm h^−1^ (7.6 ± 2.2 µm h^−1^ in the wild-type, the difference was statistically significant with *p* < 0.0001; figure 2*b*, table 1). The time period of sporogenic hyphae extension was only marginally longer in the Δ*parA* strain than in the wild-type (335 ± 85 min versus 299 ± 61 min; table 1, figure 2*c*). As a result of the higher growth extension rate and the somewhat longer extension period, the length of the sporogenic hyphae of the Δ*parA* strain at growth cessation was approximately 170% of wild-type hyphae length (61 ± 16 µm and 36 ± 13 µm, respectively, significant with *p* < 0.0001, figure 2*d*, table 1). Complementation of *parA* deletion restored the hyphal length (electronic supplementary material, figure S1*b*). Thus, our observations revealed that *parA* deletion significantly increases the length of the hyphae and the extension rate.

Interestingly, in contrast with the Δ*parA* results, the Δ*parB* strain (MD021) exhibited a hyphal extension rate decreased to approximately 70% of the wild-type strain rate (5.5 ± 2.1 µm h^−1^, *p* < 0.01) and approximately 50% of the extension rate of the *parA* deletion strain (*p* < 0.0001; figure 2*a,b*). Although the time period of hyphal extension in the Δ*parB* strain increased to 120% of the wild-type (367 ± 88 min, *p* = 0.011), the final length of MD021 hyphae did not differ significantly from the wild-type hyphae length (31 ± 11 µm, in comparison with 36 ± 13 µm, respectively; figure 2*c,d*, table 1). Thus, the effect of *parB* deletion on the hyphal extension rate is the opposite of the *parA* deletion.

If the effect of ParB on the extension rate results from the effect of ParB on ParA assembly, a double *parAB* deletion strain should exhibit similar characteristics to the Δ*parA* strain. However, the hyphal extension rate of the Δ*parAB* (MD031) strain was still 80% (6.3 ± 2.1 µm h^−1^, figure 2*b*, table 1) of the wild-type, which was similar to the Δ*parB* strain and almost two times lower than the Δ*parA* hyphal extension rate (only the latter difference was statistically significant; *p* < 0.0001). Additionally, the hyphal extension time was similar to the wild-type strain (319 ± 115 min, figure 2*c*, table 1), while the hyphal length was, on average, the same as the Δ*parB* strain (figure 2*d*, table 1). The analysis of the double deletion mutant suggests the increased extension rate caused by ParA elimination is dependent on ParB. Moreover, ParB elimination inhibited the growth rate in both the *parB* and *parAB* deletion strains, which suggests a ParA-independent mechanism.

In sum, our results revealed that ParA and ParB affect both the extension rate of the sporogenic hyphae and the period of hyphal extension. We also found that the two proteins have opposite effects on hyphal extension.

### 3.3. ParA induces the formation of massive ParB complexes during sporulation

Earlier studies of *S. coelicolor* demonstrated that regularly distributed ParB complexes are formed when ParA spreads along the sporulating hyphae [30], which is supported by our time-lapse microscopy studies of *S. venezuelae*. These results suggest that ParA accumulation along the hyphae promotes the initiation of chromosome segregation. Because we were not able to analyse the formation of ParB complexes during sporulation by time-lapse microscopy, to study their formation and dependence on ParA, we carried out ChIP-seq analysis of the wild-type and Δ*parA* strains (the Δ*parB* strain was used as a negative control). To determine the appropriate time points for the ChIP-seq analysis, we analysed the sporulation in liquid cultures and found that accumulation of FtsZ and ParA began at the 14th hour of culture, with the maximum at 16–18 h (electronic supplementary material, figures S2 and S3). As the ParB complexes are expected to form at the very early stage of ParA and FtsZ induction and remain after septation of the hyphae, we analysed ParB binding at the two time points—the 14th and 20th hour of culture growth.

**Figure 3.**
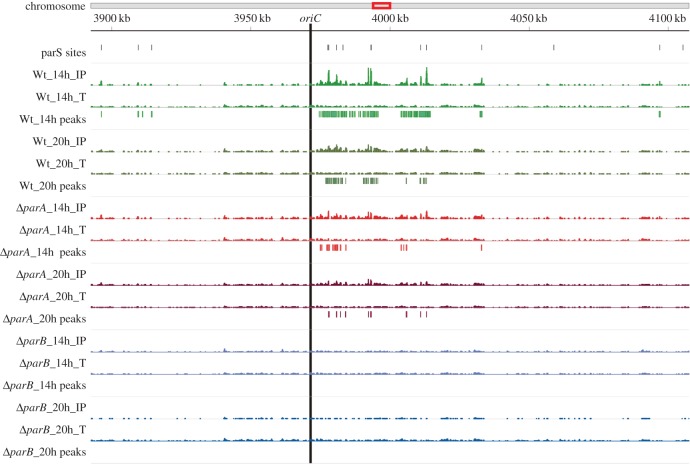
ChIP-seq analysis of ParB binding to the *S. venezuelae* chromosome. Top: the red rectangle on the chromosome indicates the enlarged region shown below. In this region, from top to bottom, there are: positions of 16 *parS* sites (with two pairs overlapping, not distinguishable due to resolution and short distance), the distribution of mapped reads for an immunoprecipitation sample (IP) and its corresponding input control of total DNA (T) as well as high-confidence peaks for the wild-type, Δ*parA* and Δ*parB* strains and the 14th and 20th hour cultures. The thick perpendicular line indicates the *oriC* region identified experimentally in *S. venezuelae* (G. Plachetka and J. Zakrzewska-Czerwińska 2013, unpublished data). The results are based on Novoalign-mapped reads. The distribution of reads is in the same scale for all tracks.

First, we searched the *S. venezuelae* chromosome for *parS* sites *in silico*. In *S. coelicolor*, 23 *parS* palindromes are located in the chromosome centre and only one near the chromosome end. In the *S. venezuelae* chromosome, we identified 16 palindromic *parS* sequences (with *p* < 0.000001). Similar to *S. coelicolor*, their localization is restricted to the central region of the *S. venezuelae* chromosomes near the *oriC* site within approximately 200 kb (figure 3).

Analysis of the binding profile confirmed that ParB bound the central region of the chromosome near the *oriC* (figure 3). Almost all identified peaks corresponded to the *parS* sites (results based on reads mapped by two aligners, Bowtie and Novoalign, were very similar). Binding was also detected between the *parS* sites, which suggests the formation of large nucleoprotein ParB complexes encompassing approximately 40 kb proximal to *oriC* (electronic supplementary material, figure S4*c*). No binding was detected in the negative control (Δ*parB* mutant, MD002). Comparison of ParB binding profiles suggests more efficient binding in the 14th hour of culture than in the 20th hour of culture (the efficiency of immunoprecipitation, e.g. formaldehyde cross-linking should not be affected by the time of culture [51]). This was reflected by the higher number of peaks identified (78 and 73 for Bowtie and Novoalign-mapped reads in the 14th hour, respectively, and 37 and 33, respectively, in the 20th hour; figure 3). In the Δ*parA* strain, the binding profile suggests less efficient ParB binding than in the wild-type strain, which was confirmed by the smaller number of peaks identified (figure 3). Additionally, the binding profile (and the number of identified peaks) in the 14th and 20th hour is very similar. It suggests that in the absence of ParA, ParB binding is diminished, is less extensive and is not enhanced at a particular time in development (14th hour) as in the wild-type strain.

In summary, ChIP-seq analysis confirmed the formation of large nucleoprotein ParB complexes encompassing *parS* sites and the regions between the binding sites at early sporulation time points. Additionally, the analysis showed that ParA is required for the formation of fully assembled ParB complexes at the early stage of sporulation, reinforcing the hypothesis that ParA accumulation facilitates formation of segrosomes.

### 3.4. Aberration of DNA segregation affects formation of Z-rings

The primary role of the ParB protein in *Streptomyces* is the formation of nucleoprotein complexes in sporulating hyphae, whereas ParA is required for their regular distribution along the hyphae and for their efficient assembly [30], which was supported by the ChIP-seq experiments. Although electron microscopy studies suggested that in *S. coelicolor,* septa may be formed over non-segregated DNA [24], our previous observations of minicompartments (delimited by closely spaced septa), which are particularly abundant in Δ*parA* and also present in the Δ*parB* strain [30], indicated that the chromosome organization affects the positioning of Z-rings. Here, using time-lapse microscopy, we determined whether chromosome missegregation resulting from the deletion of *parA* and/or *parB* affects the timing and duration of Z-ring formation.

**Figure 4.**
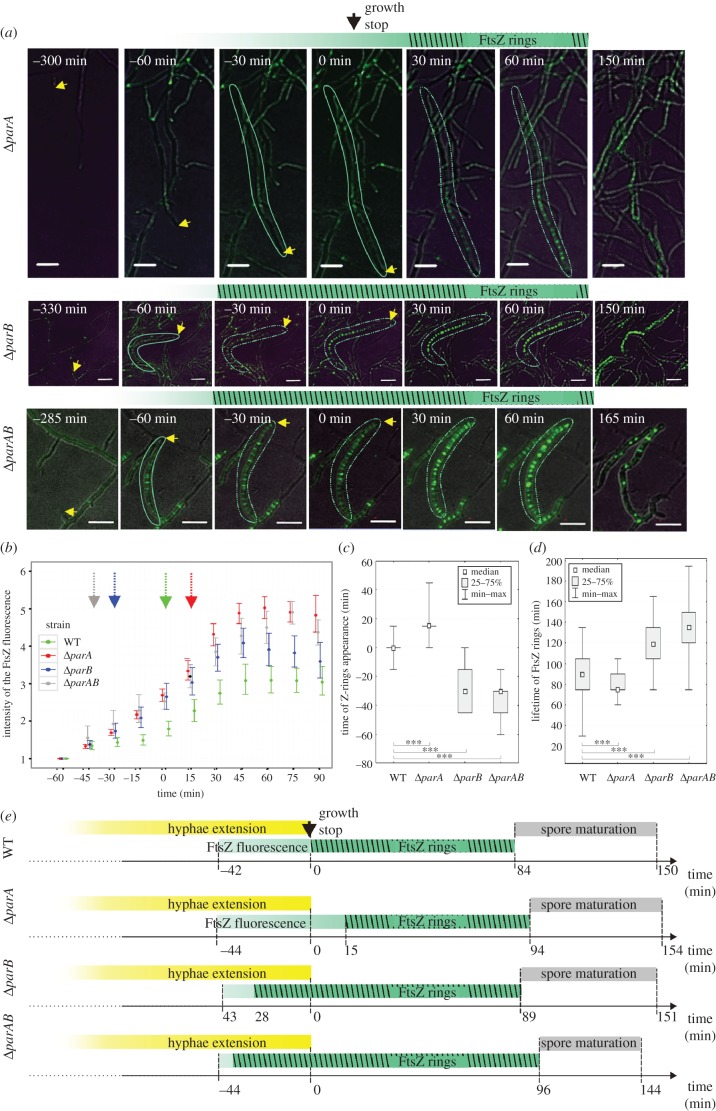
Altered timing of Z-ring formation in the Δ*parA*, Δ*parB* and Δp*arAB* strains. (*a*) FtsZ-YPet fluorescence in the wild-type, Δ*parA*, Δ*parB* and Δ*parAB* strains. The yellow arrowhead indicates the position of the growing hyphae, the green outline indicates the hyphae with an increase of FtsZ fluorescence, and the broken green outline indicates the hyphae with Z-rings. Scale bars, 5 µm. (*b*) The fluorescence intensity of the Z-rings in WT, Δ*parA*, Δ*parB* and Δ*parAB* strains. (*c*) Box plots of the time of Z-ring appearance in relation to growth cessation. (*d*) Box plots of the lifetime of FtsZ rings (asterisks indicate statistically significant results in comparison to wild-type; *p < 0.1, **p < 0.01, ***p < 0.001). (*e*) Scheme of the Δ*parA*, Δ*parB* and Δ*parAB* hyphae differentiation stages.

In the hyphae of Δ*parB* and Δ*parAB* merodiploid strains expressing *ftsZ-ypet* (MD021 and MD031), Z-rings formed significantly earlier than in the wild-type strain, on average 28 ± 15 min and 39 ± 12 min (mean ± s.d.) prior to growth cessation, respectively, while in the wild-type, the Z-rings were visible exactly at the time of growth cessation (*p* < 0.00001; figure 4*a–c*,*e*, table 2; electronic supplementary material, movie S3). Surprisingly, in the Δ*parA* strain (MD011), Z-rings were visible later, only 15 ± 12 min after growth cessation (figure 4*a–c*,*e*, table 2; electronic supplementary material, movie S4). The level of FtsZ protein was similar in all analysed strains (electronic supplementary material, figure S3), showing that the differences in timing of the Z-ring formation are not clearly related to *ftsZ* overexpression but rather result from an accelerated assembly of the Z-rings. The earlier appearance of Z-rings was associated with their significantly longer ‘lifetime’ in the *parB* and *parAB* deletion strains compared with the wild-type (117 ± 21 min in Δ*parB*, 135 ± 28 min in Δ*parAB* and 84 ± 26 min in wild-type strain, *p* < 0.00004; table 2, figure 4*d*). In Δ*parA*, the overall lifetime of Z-rings was similar to the wild-type (79 versus 84 min, respectively; figure 4*d*). No significant differences were observed between the wild-type and deletion strains in the spore maturation time, and the total time of sporulation measured from growth cessation to spore separation was similar in all analysed strains (table 2). This indicates that ParA and ParB do not influence the duration of the later stages of the *Streptomyces* cell cycle, but they visibly affect the timing of Z-ring formation.

**Figure 5.**
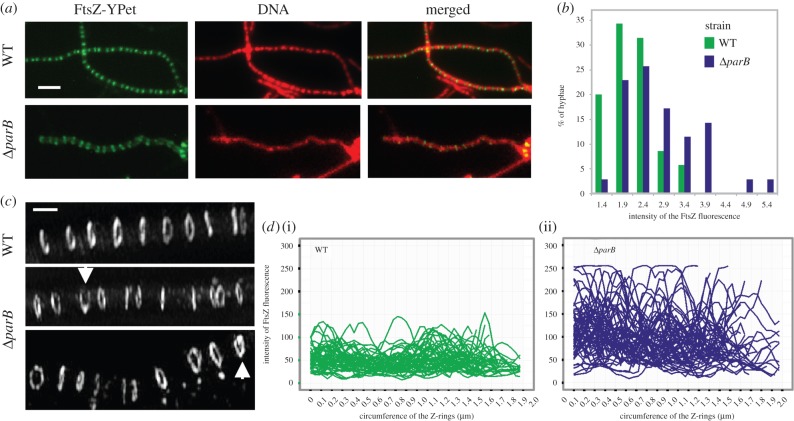
Z-ring aberrations in the Δ*parB* strain. (*a*) Examples of the hyphae with Z-rings and non-segregated DNA in the Δ*parB* strain. Scale bar, 5 µm. (*b*) Variation of the Z-ring intensity in the wild-type and Δ*parB* strains. (*c*) Representative structured illumination microscopy images of FtsZ rings in the hyphae of the wild-type and Δ*parB* strains. The white arrowhead indicates broken Z-rings. Scale bar, 1 µm. (*d*) Measurement of fluorescence intensity at each point along the circumference of the Z-rings in the wild-type (i) and *parB* deletion strain (ii) (measured for 60 rings in eight hyphae representative of various time points during septation).

In contrast with the timing of Z-ring formation, the timing of FtsZ fluorescence appearance was not significantly different in the hyphae of all analysed deletion strains and the wild-type (figure 4*b*,*e*, table 2). In all studied strains, the intensity of Z-ring fluorescence increased over time (figure 4*b*). A similar increase in overall FtsZ-YPet fluorescence was observed in the liquid culture time course (electronic supplementary material, figure S3). The deletion strains showed somewhat higher intensity of FtsZ-YPet fluorescence along the hyphae and higher intensity of Z-ring fluorescence (figure 4*b*). Notably, in the Δ*parB* strain, Z-rings along the hyphae exhibited a variation of intensity that was three times greater than in the wild strain (*p* < 0.002) at the time of growth cessation (figure 5*b*).

In the *Streptomyces* hyphae, chromosomes condense and segregate during formation of Z-rings [24,30]. Because the Z-rings form earlier in the Δ*parB* strain, prior to growth cessation, we determined if the earlier formation of Z-rings precedes DNA segregation. Indeed, in contrast with the wild-type strain where Z-rings were detected in the hyphae with condensed nucleoids, in the Δ*parB* strain, approximately 10% of hyphae with Z-rings still contained unsegregated and uncondensed nucleoids (figure 5*a*).

We next investigated whether the accelerated formation of Z-rings in the hyphae with non-segregated chromosomes in the Δ*parB* mutant was associated with any aberrations in their Z-ring structure. To compare the Z-ring structure in the wild-type and Δ*parB* strains, high-resolution structured illumination microscopy was used. In the wild-type strain, Z-rings had a regular shape and uniform intensity of fluorescence, but in the Δ*parB* strain, they were often broken or included local accumulation of FtsZ, which affected the regular shape of the structures (figure 5*c*). Measurements of fluorescence intensity along the circumference of the rings in 10 randomly selected hyphae supported this observation (figure 5*d*). In the *parB* deletion strain, the variation of the Z-ring fluorescence intensity along the Z-rings was two times higher than in the wild-type strain (proved with the *F*-test for the intensities scaled up to similar values, with the ratio of variances 1.18 ± 0.089; *p* = 0.000037). Moreover, approximately 20% of the analysed rings were broken (non-continuous) compared with 1%–2% in the wild-type strain (figure 5*c*). These observations suggest that the elimination of ParB promotes earlier polymerization of FtsZ, but the ring assembly is not uniform.

Overall, our analyses revealed that ParB elimination promotes earlier formation of Z-rings, but these rings are less uniform than those formed in the wild-type. In the absence of ParA, the Z-rings are delayed. This may be associated with reduced ParB complex formation in the absence of ParA. Our observations suggest that ParB complexes and/or DNA organization directly or indirectly affect the assembly of Z-rings.

### 3.5. Increased chromosome compaction induces Z-ring formation

We previously observed that increased chromosome supercoiling induced by TopA depletion in *S. coelicolor* led to the inhibition of chromosome segregation and septation, while a moderate increase in chromosome compaction affected the positioning of the septa [31]. Here, our *S. venezuelae* studies showed that chromosome segregation affects the timing of sporulation septation. Thus, we tested whether the altered chromosome supercoiling resulting from decreased TopA disturbed the formation of Z-rings. The *S. venezuelae* TopA-depleted strain (MD013, in which the *topA* gene is under the control of the inducible p_tet_promoter) exhibited growth inhibition and sporulation blockage when cultured without an inducer, as previously described for the *S. coelicolor* strain [31]. Time-lapse microscopy of an MD013 derivative expressing the *ftsZ-ypet* gene (AKMD2 strain) confirmed that at the lowest level of TopA (no inducer added), the formation of Z-rings was completely abolished.

**Figure 6.**
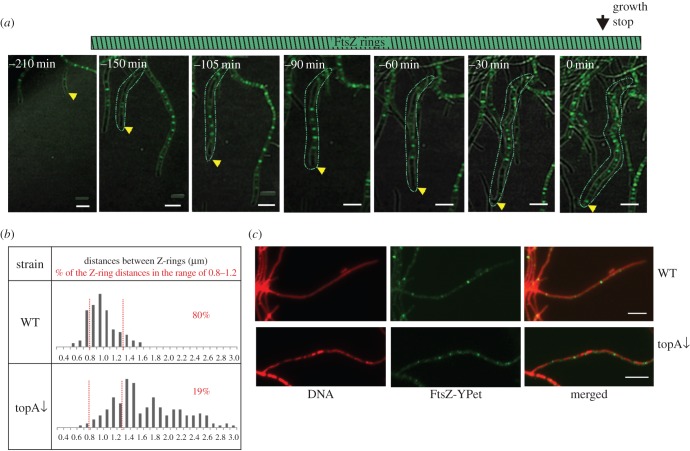
Accelerated Z-ring formation in the TopA-depleted strain. (*a*) Images of sporulating hyphae in the TopA-depleted strain (time lapse). FtsZ-YPet fluorescence (green) merged with the DIC image. Yellow arrowhead indicates the position of the growing hyphae, and the green contour indicates the hyphae with FtsZ rings. Scale bars, 5 µm. (*b*) Distances between Z-rings at the time of growth cessation in the wild-type strain and the TopA depletion strain, (*c*) images of the Z-rings (green) in DNA stained (red) sporulating hyphae of the wild-type and TopA-depleted strains. Scale bars, 5 µm.

Surprisingly, partial depletion of TopA (protein level eight times lower than the wild-type level, electronic supplementary material, figure S4*a*) led to accelerated formation of the Z-rings, which were already detectable in the extending hyphae (figure 6*a*). The Z-rings were visible more than 3 h before growth cessation, and their duration was significantly increased compared with the wild-type strain (205 ± 52 min and 84 ± 26 min, respectively, table 2). Interestingly, the time between the arrest of hyphal extension and spore separation was shortened to only about one quarter of the wild-type sporulation time (table 2). Additionally, Z-rings at growth cessation were very irregularly spaced, and they formed between the irregularly spaced regions of condensed DNA (figure 6*b,c*). Our observations suggest that the increased chromosome condensation in TopA-depleted strains promotes Z-ring formation in chromosome-free regions.

To summarize, the analyses of the strain with the modified topoisomerase levels suggest that chromosome organization directly influences the FtsZ polymerization in the ring structure. Our data also suggest that the increased chromosome compaction promotes Z-ring formation.

## 4. Discussion

In various bacterial species, ParAB proteins synchronize chromosome segregation with other cell-cycle events, such as cell division, cell elongation or chromosome replication [9,12,52]. Thus, the disruption of the *parAB* genes results in a plethora of phenotypes ranging from disturbances in chromosome segregation to variation of the cell length to chromosome over-replication and inhibition of cell division. Here, we determined how the ParAB proteins coordinate the cell-cycle events during *Streptomyces* sporulation, when tens of chromosomes are distributed evenly along every sporogenic cell.

The cessation of hyphal growth coincides with the formation of Z-rings and the accumulation of ParA. The time of induction of *parA* and *ftsZ* expression suggests their involvement in a developmental checkpoint between hyphae extension and growth cessation. This type of checkpoint was suggested earlier by the analysis of *S. coelicolor* developmental mutants with long and unseptated hyphae, *whiA* and *whiB* [50]. Previously, both the *parAB* p2 and *ftsZ* p2 promoters were shown to be developmentally regulated by the WhiA and WhiB proteins [28,29]. Our time-lapse analysis confirmed that the initiation of septation and chromosome segregation is synchronized with hyphal growth cessation.

Elimination of ParA increased the rate of hyphal growth approximately 1.5 times and led to increased hyphae length at growth cessation and longer spore chains. Elongated spore chains were observed before in *S. coelicolor*, and this phenomenon was explained by the interaction of ParA with Scy, a tip-localized component of the polarisome complex (or TIPOC, tip organizing complex) responsible for hyphae elongation [11,20,32,53]. According to our model, cell extension is promoted when the balance between ParA and Scy is shifted towards Scy assembly. The induction of *parA* expression in sporulating hyphae is associated with the disassembly of Scy at the tip, which triggers the cessation of hyphal elongation and commencement of sporulation [11]. Interestingly, ParA signals in *S. venezuelae* could be detected only as diffuse fluorescence along the hyphae, but tip localization was not clearly detectable, which could possibly be due to a different experimental set-up or a weak transient ParA signal. The strict coordination of the ParA induction and growth cessation observed in *S. venezuelae* suggests that in this species, ParA is also engaged in the developmental checkpoint.

Surprisingly, time-lapse analyses showed that elimination of ParB decreases the hyphal growth rate. However, the hyphal extension time of the *parB* deletion strain was increased, allowing the hyphae to reach a length approximately similar to the wild-type hyphae. This suggests the presence of a checkpoint mechanism that is based on control of the hyphal length rather than the extension time. Such observation is reminiscent of recently reported constant size extension which was suggested to maintain size homeostasis in *E. coli* and *C. crescentus* [54]. The opposite effect of *parB* deletion and *parA* deletion on hyphal extension could be explained by the modulation of ParA ATPase activity, its nucleotide exchange or ability to interact with Scy by ParB. If that was the only mechanism accounting for the observed changes in growth rate, the hyphal extension of the double *parAB* deletion strain should be similar to the *parA* deletion strain. Interestingly, the double *parAB* mutant exhibited a decreased rate of hyphal extension, which suggests the involvement of ParB in an additional, ParA-independent mechanism(s) influencing growth. One possible mechanism could be the influence of ParB on chromosome replication or transcription of the genes in the region involved in complex formation, which could affect the rate of hyphae growth. The other possibility is the engagement of ParB with the polarisome (TIPOC) complex. A direct interaction between ParB and DivIVA has been suggested for *Corynebacterium glutamicum*, which also belongs to the Actinobacteria phylum and exhibits a polar mode of growth [55]. However, the relatively far distance from the ParB complex to the tip in *Streptomyces* hyphae (1.4 µm) challenges the hypothesis of direct interaction. Interestingly, in *C. glutamicum*, the reduced rate of polar cell elongation was caused by *parA* deletion [56]. It is difficult to account for the contrasting effects of *parA* and *parB* deletion in *Streptomyces* and *Corynebacterium*, but presumably, during the transition from the rod cell polar extension to hyphal growth, a new mechanism(s) controlling cell elongation evolved. Although at present we cannot provide an explanation for the observed ParB influence on the hyphal extension, our studies revealed that the hyphal elongation rate in *Streptomyces* is controlled by a not yet fully identified mechanism that involves both segregation proteins.

In *S. coelicolor*, ParA spreads from the tip complex to an elongated structure and was suggested to trigger formation of regularly spaced ParB complexes along the hyphae. We showed that ParB-EGFP foci in the sporulating hyphae of the *parA* deletion strain are irregular compared with the wild-type and showed much weaker fluorescence [30]. The ChIP-seq analysis confirmed that ParB complexes encompassed large chromosomal fragments. It was suggested that ParB homologues are able to trap DNA loops by DNA bridging or binding DNA near *parS* over long distances [57,58]. Our findings are consistent with the observation made in *B. subtilis* that the ParB complex engages a chromosomal region of several kilobases in length. The presence of ParA stimulates the formation of large nucleoprotein complexes in *S. venezuelae* and initiates chromosome segregation.

The time-lapse analysis revealed that ParAB proteins affect the formation of Z-rings. The earlier Z-ring formation in the *parB* and *parAB* mutant strains could not be directly related to the earlier increase in FtsZ levels. However, in the absence of ParB, the Z-rings had very irregular shapes and uneven fluorescence intensity. This suggests that either chromosome organization or ParB itself affects the assembly of the Z-rings. The effect of *parAB* deletion on the septation has been noted before, as has the uneven spacing between the septa in the *parA* and *parB* mutant strains [30]. Interestingly, the disturbances in chromosome segregation and septation observed in *S. venezuelae parAB* deletion strains were less severe than those described earlier for *S. coelicolor* [30]. This may result from either differences in growth characteristics between the species or the presence of plasmid-encoded segregation proteins (*S. venezuelae* genome contains plasmids with *parAB* homologues, but nothing is known about their expression) that complement the *parAB* deletion as observed earlier in the case of the *S. coelicolor* SCP1 plasmid [59]. In contrast with Δ*parB*, *parA* deletion delayed initiation of septation. In the Δ*parA* strain, ParB still formed complexes, although not efficiently, which remained irregularly spaced. The observed delay in Z-ring assembly in the Δ*parA* strain suggests the direct involvement of ParB complexes in control septation.

Notably, the inhibitory effect of unsegregated chromosomes and ParB complexes on the septation was also suggested by our studies of a topoisomerase I (TopA)-depleted strain [31]. ChIP-seq analyses confirmed ParB binding to most of the *parS* sites in the TopA-depleted strain, although the binding profile differed from the one observed in the wild-type strain (electronic supplementary material, figure S4*b*). The other explanation for early Z-ring formation in the TopA depletion strain, which cannot be excluded, is that increased chromosome supercoiling induces transcription of some regulators that control septation. Our studies of *S. venezuelae* showed that moderately increased chromosome supercoiling promotes Z-ring assembly even in extending hyphae. We observed that the intensity and spacing of the Z-rings in the TopA-depleted strain at the time of growth cessation were very irregular. This also suggests that their position may be determined by irregular condensation of the chromosome.

Our observations suggest the involvement of the ParB protein in negative control of Z-ring formation. In fact, in some bacterial species, the ParAB proteins are involved in septum placement through interaction with regulators of FtsZ polymerization, such as MipZ in *C. crescentus* [12] or PomZ in *Myxococcus xanthus* [60]. MipZ (a ParA family protein), through interaction with ParB, forms a gradient extending from the pole and inhibits the formation of Z-rings close to the pole. Contrary to MipZ, PomZ (another ParA family protein) was suggested to positively regulate division site selection by recruitment of FtsZ. Interestingly, in *B. subtilis,* Noc is a ParB homologue that interacts with the chromosome and cell membrane [61]. In *Streptomyces*, neither potential FtsZ regulators interacting with ParB nor other ParB homologues that could act as NO proteins were identified [21]. To date, the only described mechanism controlling septa placement is the positive control by Ssg proteins [25]. SsgA and SsgB were detected prior to Z-ring formation at the aerial hyphae membrane, and SsgB is speculated to tether the FtsZ filaments [62]. Because an *ssgB* mutant strain also showed impaired growth cessation [63], it is plausible that the ParAB proteins are involved in the interaction with Ssg proteins. Interestingly, the increased variation of septa placement in *parAB* deletion mutants has been observed before in other bacteria, such as *M. smegmatis* or *C. glutamicum* [10,56]. For those species, it was also suggested that the nucleoid itself or the interaction with an unknown factor determined the positioning of the Z-rings [18]. Consistent with this idea, we suggest that *Streptomyces*, in the absence of nucleoid occlusion proteins (Noc or SlmA), developed another negative control mechanism to spatially and temporally regulate septum formation.

**Figure 7.**
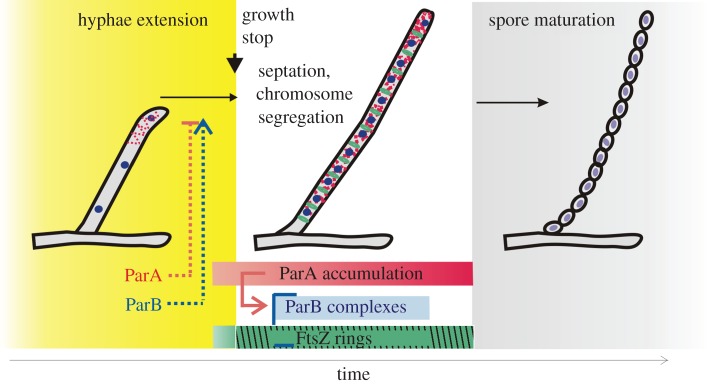
Model showing the involvement of segregation proteins in the hyphae extension/septation checkpoint and the close link between ParB complexes and Z-ring formation at the time of growth cessation.

In summary, we demonstrated that the function of ParAB in *Streptomyces* extends from chromosome segregation to coordination of this process with cell elongation and cell division (figure 7). During *Streptomyces* sporulation, hyphae elongation ends before chromosome segregation and septation. The unique *Streptomyces* spatio-temporal separation of cell elongation and division imposes special requirements for synchronization checkpoints on both processes. Our data show that ParAB proteins are involved in these checkpoints and coordinate the transition from hyphal growth to segregation of tens of chromosomes associated with septation. The formation of ParB complexes determines the timing of septation. Moreover, we demonstrate that the absence of ParB affects the assembly and distribution of Z-rings. Thus, our analysis shows a close link between Z-rings and ParB complexes.
